# From Pathways Databases to Network Models of Switching Behavior

**DOI:** 10.1371/journal.pcbi.0030152

**Published:** 2007-09-28

**Authors:** Baltazar D Aguda, Andrew B Goryachev

**Affiliations:** Whitehead Institute, United States of America

## Introduction

The excitement in today's biology is driven by the huge amounts of information generated by high-throughput data-acquisition technologies, and by the expectation that these datasets will soon provide detailed understanding of life's processes. Ultimately, these datasets have to be integrated into a framework that facilitates the study of the dynamics arising from networks of physico–chemical interactions orchestrating the physiology of a biological cell. The bioinformatics community is actively responding to this call for integration in terms of frameworks of pathways databases [1,2]. This paper addresses the use of these databases as sources of dynamical models for biological phenomena. We focus here on models that are based on molecular interactions and how these interactions are coupled to explain observed cellular behavior. The model-building process that we describe below takes the point of view of a non-biologist who has access to online pathways databases but has not been directly involved in relevant experimental studies. Of course, one could argue that a better approach is for the modeler to collaborate with a biologist who is already familiar with the system and has developed intuition about how it works; in other words, the biologist may already have a “model” in mind—usually called a “hypothesis”—and what remains to be done is to encode this model in the language of mathematics. Note that this “hypothesis-driven” modeling approach already assumes a reduced network in the beginning of the modeling process. In contrast, we would like to show in this paper how a reduced network model is extracted from a much larger network, given a specific biological question and a set of relevant experimental observations.

Mathematical models range from qualitative and probabilistic models to quantitative and deterministic kinetic models [3–5]. The chosen set of molecular interactions and processes form what we call a “network model.” Although the networks can have various degrees of detail, they all have the common property of being composed of nodes and edges representing interactions between nodes. Definitions of networks and pathways, as well as an example of a network model are given in the next section. Ultimately, we are interested in mechanistic models with well-defined molecular interactions or reaction mechanisms and corresponding rate equations that are subsequently solved numerically to simulate the phenomenon. Although mechanistic details are becoming available in increasing numbers of online pathways databases and knowledgebases, quantitative values of most kinetic parameters are still lacking—and this problem is compounded by the fact that many details of these pathways can be cell-specific (with regard to cell type, organism, etc.) and can have variability even among the same cell type in an organism. We provide below an overview of pathway resources on the Internet. Note that we are not concerned here with computational methods of determining or inferring network topology, or connectivity from *omics* data—on this topic the reader is referred to an article of Qi and Ge [3] that appeared in this journal recently.

In this article, we illustrate how one extracts a reduced network model from a large preliminary network obtained from databases. The model extraction procedure is explained in the context of a specific biological *question* about a cell cycle checkpoint called the “restriction point” (R point)—that is, what is the smallest subset of interactions in the given network that can account for the switching behavior associated with this checkpoint? A method of qualitative network analysis is proposed to zoom into a core subnetwork which accounts for the essential qualitative behavior being modeled. Once the core network model is established, a kinetic model is constructed and a suite of mathematical analysis and computer programs can be used for further investigation.

## Qualitative versus Mechanistic Network Models

We limit our definition of a *network* to a connected graph composed of nodes and edges. An edge connects at most two nodes, and the connectedness of the graph means that there exists at least one continuous path (regardless of edge direction) linking any two nodes in the network. A node can represent physical entities (genes, ions, molecules, protein complexes, etc.) or modules (defined to include processes or subnetworks with identifiable functions, but whose internal details are not shown in the graph; for example, the mechanistic details of a signaling module can be hidden if we are merely interested in the module's input–output response to external signals). Thus, a qualitative network model presumes that the set of nodes and edges is sufficient to describe the phenomenon of interest; if it is a dynamical phenomenon, then dynamical variables are associated with the nodes, and rates of transitions from one variable to another are associated with the edges.

**Figure 1:**
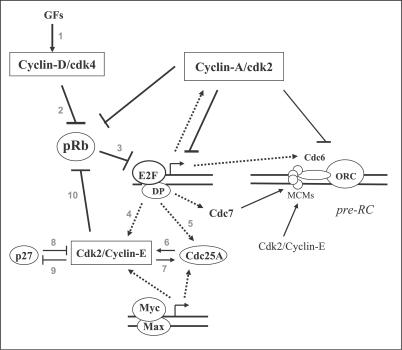
The Regulatory Network of the G1-S Transition in the Mammalian Cell Cycle Growth factors (GFs) trigger certain signaling cascades that lead to the activation of cyclin D/CDK4 complexes. Active CDK4 phosphorylates (thereby deactivating) the retinoblastoma protein (pRb) which inhibits entry into S phase due mainly to inhibitory binding with E2F transcription factors; these factors induce many of the genes required for S phase (such as members of the pre-replication complex, cyclin E, cyclin A, Cdc25A, etc.). Synthesis of cyclins E and A leads to activation of CDK2 which further phosphorylates (thereby deactivates) pRb. Another transcription factor, namely Myc, also contributes to the G1-S transition, but this protein's regulation is not shown. Arrows mean “activate,” and hammerheads mean “inhibit.” The dashed arrows signify the totality of gene expression steps (transcription and translation). Interactions numbered 1 to 10 form a minimal model that can account for the R point behavior.

An example of a network graph representation commonly encountered in the molecular biology literature is shown in Figure 1. The edges in the graph are all directed binary interactions. The graph is referred to as a qualitative network (*qNET*) because only the qualitative nature of the binary interactions is depicted (an arrow means “activates” and a hammerhead means “inhibits”).

**Figure 2:**
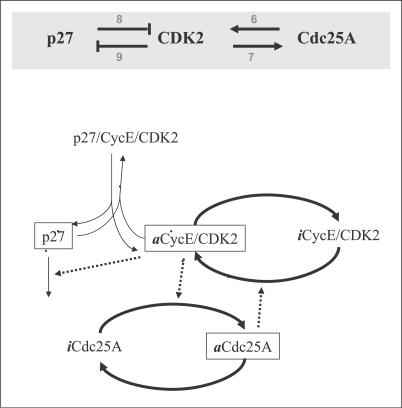
A Sharp Switch Is Expected from the Mutual-Activation and Mutual-Inhibition Topology Involving CDK2 Also shown are the known detailed mechanistic steps corresponding to the qNET shown in the upper panel (shaded grey). “a” refers to active, and “i” to inactive. Note that the network in the lower figure uses the chemist's convention of representing reaction steps; also, dashed arrows mean that the protein where the arrow originates from induces or catalyzes the reaction step that the arrow points to.

If further details are known about the interactions, one can transform the *qNET* into a mechanistic network model. An example is illustrated in Figure 2 where the four qualitative interactions among three nodes (upper panel) are shown to correspond to seven mechanistic steps involving six proteins or protein complexes (lower panel). In general, existing online pathways databases contain mixtures of qualitative and mechanistic interactions. In formulating the dynamical equations of a network model having such mixtures of interactions, standard chemical kinetic theory is used for the rates of interactions with known mechanisms while phenomenological equations (i.e., functional representations of activation and inhibition) are used for the qualitative interactions. An example of a phenomenological rate equation representing the observation *Y is inhibited by X* is d*Y*/d*t* = *k*_1_/(*k*_2_+*X^n^*), where *X* and *Y* are concentrations, *k*_1_ and *k*_2_ are constants, and *n* could be an adjustable parameter of the model.

## Preliminary Network Models from Databases

**Figure 3:**
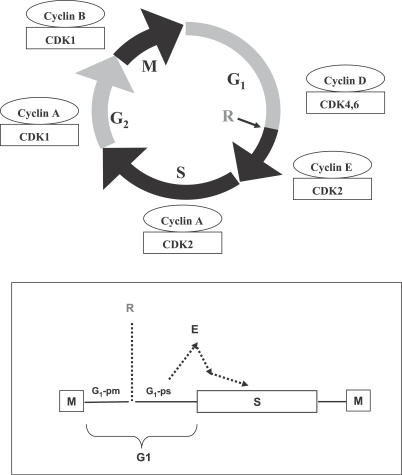
The Mammalian Cell Cycle Showing the G1, S, G2, and M Phases along with the Predominant Cyclin–CDK Activities Associated with Each Phase (Top Panel) The lower panel shows the position of the R point (R) which subdivides the G1 phase into G1-pm (post-mitosis) and G1-ps (pre-S-phase). Quiescent or non-dividing cells have to be exposed to continuous growth-factor stimulation up until the R point in order to commit to entry into S-phase. After R and a finite induction period, cyclin E/CDK2 activity increases (shown by the dashed curve labelled E) as reported by Ekholm et al. [6].

In this and the next section, we illustrate how one can extract a network model of the *restriction point* (R point) in the mammalian cell cycle. The R point is a checkpoint in mid to late G_1_ phase [6,7]. It is considered as the point of commitment to replicate the DNA where it is “sensed” that the prerequisites for cell cycle progression are satisfied (e.g., sufficient cell size and undamaged DNA). The modeling goal is to explain the mechanistic and kinetic origins for an observed switching behavior associated with the R point. This switch pertains to the experimentally observed activation of cyclin E/CDK2, as reported by Ekholm et al. [6] (see Figure 3). In the model-extraction procedure discussed below, we will refer to growth-factor signaling pathways as the “processes” that induce the R point switch and to cyclin E/CDK2 as the “marker” for the switch.

The first step in building a network model is to identify the nodes of the network. It would be easy to use literature reviews written by specialists on the topic, but, as we mentioned earlier, we start afresh by using information taken from online databases. Since R point regulation is embedded in the G1-S regulatory network, one may start by visiting the *Gene Ontology* (GO) Web site (see Table 1) to obtain a list of annotated genes. GO's classification and hierarchy of *biological processes* can be used as a starting point for identifying the parts list of the G1-S molecular machinery. An example of a GO search sequence, using the Amigo browser, is the following: under the category of *biological processes,* click on *cellular process* followed by *cellular physiological process, cell cycle, regulation of cell cycle,* and *cell cycle checkpoint*. Another recommended database is *KEGG* (Kyoto Encyclopedia of Gene and Genomes, see Table 1). Within a network hierarchy provided in *KEGG BRITE,* one finds (under KEGG pathway maps) *cellular processes,* which links to *KEGG PATHWAY,* containing manually curated pathway diagrams of the cell cycle. For more details on the mechanisms, one can peruse *Reactome* (see Table 1), which is a knowledgebase of human biological pathways. Sources of network diagrams for specific functional modules contributed by members of the research community at large include *GenMAPP, Biocarta,* and *PID* (see Table 1). We view these contributions as elements of a growing *library of pathway modules*. One may find redundancies, inconsistencies, incompleteness, and other sources of uncertainties in the contributed modules in these databases. Nevertheless, it is useful to view these contributed modules as preliminary models themselves for computational biologists to investigate further.

**Table 1:**
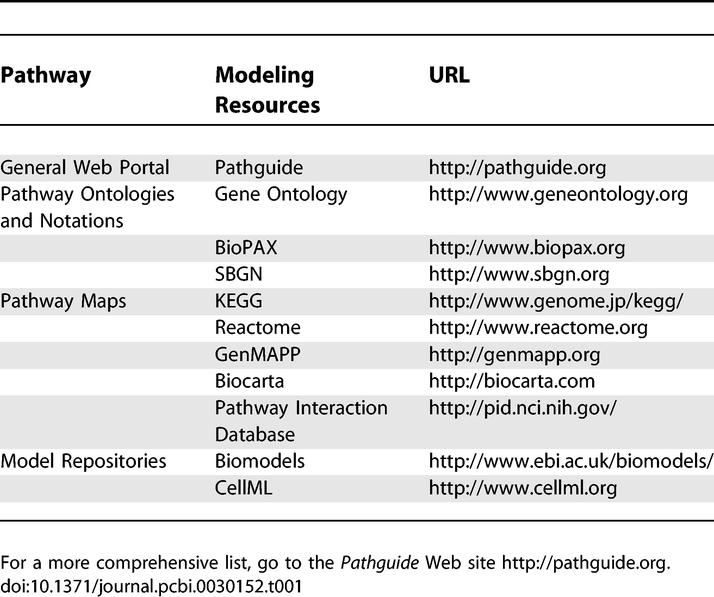
A Few Major Pathway and Modeling Resources on the Internet

A comprehensive Internet portal on pathway resources is provided by *Pathguide* (see Table 1), which links to more than 200 Web sites containing a wide range of information from pathway components (e.g., protein–protein interactions) to integrated pathway diagrams. A welcome activity in bioinformatics is the ongoing development of standards for pathway data representation by a biological pathways exchange consortium called *BioPAX* (see Table 1).

## Extracting a Network Model

The following steps are sufficient to extract a network model of the R point. (i) Start with an *initial qNET* large enough to subsume the network model of interest. (ii) Identify destabilizing cycles that involve the set of markers and processes. The meaning of “destabilizing cycle” will be given below. This step is required to find an instability that is assumed to be the cause of the switching behavior in the activity of cyclin E/CDK2. (iii) A network model is formed from the destabilizing cycles involving the marker and other interactions encompassing the process involved. (iv) From the network model, a kinetic model is generated using available information on the mechanisms and rate expressions for the interactions involved.

Step (i) requires knowledge of a set of biological markers and processes associated with the phenomenon to be modeled. For the R point, the *initial qNET* is given in Figure 1. (To simplify the discussion, we have not included in Figure 1 many other nodes and interactions that can be found in pathways databases.) As shown in Figure 3, the marker for crossing the R point is taken to be cyclin E/CDK2 and the process is that of growth-factor stimulation leading to the activation of the marker. For step (ii), one has to make an assumption as to what the “arrows” and “hammerheads” mean. We will define the interaction {X_j_ → X_i_} to mean ∂[*dx_i_*/*dt*]/∂*x_j_* > 0, i.e., X_j_ “activates” X_i_ because *dx_i_*/*dt* increases if *x_j_* increases (the lowercase *x*'s are the concentrations or activities). Similarly, the interaction {X_j_ –| X_i_} means ∂[*dx_i_*/*dt*]/∂*x_j_* < 0, i.e., X_j_ “inhibits” X_i_ because *dx_i_*/*dt* decreases if *x_j_* increases. These interpretations of the qualitative interactions imply that a qNET graph corresponds to a Jacobian matrix **M** (whose element *m_ij_* is equal to ∂[*dx_i_*/*dt*]/∂*x_j_*. Thus, a qNET graph gives only the algebraic signs of the elements of **M**. From **M** one can perform a stability analysis of the linearized network dynamics. The (stability) eigenvalues λ of **M** indicate whether the steady state of the system is locally stable or not (it is unstable if any eigenvalue has a positive real part). Note that only cycles in the qNET graph affect the linear stability of the network; to prove this statement (see also the appendix of [8]), one notes that the eigenvalues are the roots of the characteristic polynomial, which can be written as follows (for *n* × *n* matrix **M**):

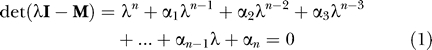
where **I** is the *n* × *n* unit matrix. The coefficients *α*_i_ in the characteristic polynomial above can be expressed as follows:

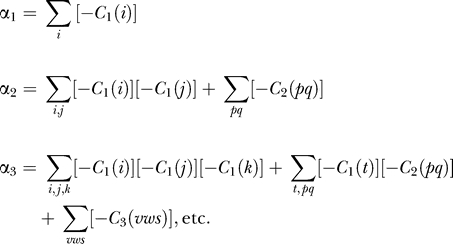
where *C_k_* is a *k*-cycle in the qNET graph; examples of these cycles for *k* = 1, 2, 3 are: *C*_1_(*i*) = *m_ii_*, *C*_2_(*pq*) = *m_pq_m_qp_*, and *C*_3_(*vws*) = *m_vw_m_ws_m_sv_*. The value of a *C_k_* is also referred to as the strength of that *k*-cycle. A cycle is said to be destabilizing if increasing its strength leads to increasing the real part of at least one eigenvalue toward the positive direction.

Carrying out step (ii) above on the network shown in Figure 1, one only considers the destabilizing cycles that involve the marker cyclin E/CDK2—and these are the following four positive loops (numbers are interaction numbers): {3, 4, 10}, {6, 7}, {8, 9}, and {3, 5, 6, 10}. In step (iii) above—which takes the aforementioned destabilizing cycles, as well as the growth-factor signaling process represented by steps 1 and 2—one arrives at a network model that is equivalent to the previously published model of [7]. The network model is thus composed of edges numbered 1 to 10 in Figure 1. There are several destabilizing cycles in this network model. Of interest is the instability due to phosphorylation–dephosphorylation cycles involved in the positive feedback interaction between Cdc25A and cyclin E/CDK2 [7,9,10]. It was shown that the switching point due to the positively coupled cycles is reached only after the levels of the proteins involved have grown above certain threshold values. Finally, step (iv) gives rise to the detailed kinetic model of [7] that reproduces the switching behavior of cyclin E/CDK2 shown in the experiment depicted in Figure 3. The simulations presented in [7] reproduce the induction period (when cyclin E/CDK2 activity is very low) after the R point, followed by the sudden increase in cyclin E/CDK2 activity; more importantly, the model simulations also show that cutting off growth-factor stimulation after the R point does not prevent the activation of CDK2.

## From Network Models to Mathematical Models

Simulation of the network model of the R point required the formulation of a system of coupled kinetic equations that can then be solved to determine the dynamics of the biological system [7]. The use of ordinary differential equations (ODEs) with the R point model is possible due to mechanistic details found in databases and the literature. However, when the interactions in a network model are poorly defined, simulation methods other than ODEs are employed. Comprehensive reviews of these methods are already available (for examples, see [11–13]) and will not be repeated here. These methods include stochastic simulations (applicable when very few molecules are involved) and Boolean dynamic simulations (applicable, for example, to a network of genes that turn each other “on” or “off”). The translation of network models to mathematical models is facilitated by the use of XML-based languages such as *SBML* (systems biology mark-up language) and *CellML* (see Table 1). Further development of *SBML* [14] is under way to provide support for storing and automatic generation of the graphical network information necessary to describe a model in mathematical terms. A parallel effort by the *Systems Biology Graphics Notation* (SBGN) consortium (see Table 1) is the ongoing development of a universal graphical notation for representation of various kinds of interaction networks. Also of interest to the modeling community is the creation of model repositories such as *Biomodels* and *CellML* (see Table 1) for networks that have matured into quantitative kinetic models.

## Concluding Remarks

The main goal of this article is to illustrate the idea that network models can be extracted from pathways databases in a systematic way. Using a specific biological phenomenon, namely the R point in the cell cycle, the modeling task is to explain the origin of the switching behavior of a protein marker when a quiescent cell is exposed to sufficient growth-factor stimulation. A large network of molecular interactions and signaling pathways is integrated from various pathways databases. Despite the lack of quantitative kinetic parameters associated with almost all of the interactions, we demonstrated that the form of qualitative network analysis described here can identify key feedback cycles in the network with potential for instability (the ultimate cause of the switching behavior). The set of these cycles is the basis for the reduced qualitative network model. Computer simulations using the final kinetic model [7]—which includes known mechanistic details—validate the prediction of a switching behavior by the model. For another detailed example of the application of the modeling approach discussed in this paper, the reader is referred to a recent work of Wee and Aguda [15] on the network of interactions between the tumor suppressor protein p53 and the oncoprotein Akt; here, the predicted switching behavior between pro-apoptotic and pro-survival cellular pathways is based on the presence of destabilizing cycles in the network. 

## Abbreviation

R point: restriction point
